# Risk factors for poor virological outcome at 12 months in a workplace-based antiretroviral therapy programme in South Africa: A cohort study

**DOI:** 10.1186/1471-2334-8-93

**Published:** 2008-07-16

**Authors:** Katherine L Fielding, Salome Charalambous, Amy L Stenson, Lindiwe F Pemba, Des J Martin, Robin Wood, Gavin J Churchyard, Alison D Grant

**Affiliations:** 1 London School of Hygiene and Tropical Medicine, London, UK; 2 Aurum Institute for Health Research, Johannesburg, South Africa; 3 Kimera Solutions, Johannesburg, South Africa; 4 University of Pretoria, South Africa; 5 Desmond Tutu HIV Centre, Institute of Infectious Disease and Molecular Medicine, Faculty of Health, University of Cape Town, South Africa; 6 CAPRISA, University of KwaZulu Natal, South Africa

## Abstract

**Background:**

Reasons for the variation in reported treatment outcomes from antiretroviral therapy (ART) programmes in developing countries are not clearly defined.

**Methods:**

Among ART-naïve individuals in a workplace ART programme in South Africa we determined virological outcomes at 12 months, and risk factors for suboptimal virological outcome, defined as plasma HIV-1 viral load >= 400 copies/ml.

**Results:**

Among 1760 individuals starting ART before July 2004, 1172 were in follow-up at 12 months of whom 953 (81%) had a viral load measurement (median age 41 yrs, 96% male, median baseline CD4 count 156 × 10^6^/l). 71% (681/953) had viral load < 400 copies/ml at 12 months. In a multivariable analysis, independent predictors of suboptimal virological outcome at 12 months were <1 log decrease in viral load at six weeks (odds ratio [OR] 4.71, 95% confidence interval [CI] 2.56–8.68), viral load at baseline (OR 3.63 [95% CI 1.88–7.00] and OR 3.54 [95% CI 1.79–7.00] for 10,001–100,000 and >100,000 compared to <= 10,000 copies/ml, respectively), adherence at six weeks (OR 3.50 [95% CI 1.92–6.35]), WHO stage (OR 2.08 [95% CI 1.28–3.34] and OR 2.03 [95% CI 1.14–3.62] for stage 3 and 4 compared to stage 1–2, respectively) and site of ART delivery. Site of delivery remained an independent risk factor even after adjustment for individual level factors. At 6 weeks, of 719 patients with self-reported adherence and viral load, 72 (10%) reported 100% adherence but had <1 log decrease in viral load; conversely, 60 (8%) reported <100% adherence but had >= 1 log decrease in viral load.

**Conclusion:**

Virological response at six weeks after ART start was the strongest predictor of suboptimal virological outcome at 12 months, and may identify individuals who need interventions such as additional adherence support. Self reported adherence was less strongly associated but identified different patients compared with viral load at 6 weeks. Site of delivery had an important influence on virological outcomes; factors at the health system level which influence outcome need further investigation to guide development of effective ART programmes.

## Background

An unprecedented effort by global organisations, governments and health care workers has achieved access to antiretroviral treatment (ART) for 2.0 million HIV-infected individuals in low- and middle-income countries by December 2006. Despite these efforts, 72% of the estimated 7.1 million who need ART globally are not on treatment, and hence strategies to improve access to care for HIV-infected persons have been the subject of much discussion [1,2]. As more patients start ART, treatment programmes face the emerging challenge of achieving and maintaining virological suppression in increasing numbers of patients for sustained periods. Treatment success requires excellent adherence to ART, which is difficult to maintain [3]. A suboptimal virological response to ART may lead to increased transmission of ART-resistant virus strains and ultimately to a dramatic reduction of the therapeutic lifespan of available ART regimens [4-6].

ART programmes in developing countries have reported varied treatment outcomes. Pilot projects established by non-governmental organisations in South Africa and Haiti have reported good virological results [7-9] although others have been less encouraging [10-12]. Adherence to ART is a major determinant of virological outcome [3,13,14] and in industrialized countries other baseline factors influencing virological response have included disease stage [15], viral load [15,16], and previous exposure to ART[15], although associations with baseline CD4 count and viral load are not consistent [17,18]. Determinants of virological outcomes, and the effect of differences in the health delivery system on outcomes, have not been studied in detail in resource limited settings. Understanding these issues is important to guide the development of effective ART programmes.

Another obstacle to effective HIV care in resource-limited countries is limited access to laboratory facilities to monitor the virological and immunological markers that are considered the standard of care in wealthy nations. For middle-income settings, where there is limited laboratory support, data are needed to inform guidelines on how best to use scarce resources for laboratory monitoring; for example to decide at what point after starting ART it is most useful to measure viral load to ensure the best outcomes for patients.

We report risk factors for suboptimal virological outcome at 12 months among HIV-infected adults receiving ART in a workplace-based HIV care programme in South Africa.

## Methods

### ART clinic protocol

This workplace-based HIV care programme, primarily serving the mining workforce, has been described in detail elsewhere [19]. The programme was designed by Aurum Institute and has been implemented at workplace sites in Southern Africa, which vary from large hospital clinics through occupational health centres to general practitioners' offices. Standard guidelines were developed for the programme and are used at all sites; these provide guidance on all aspects of HIV care including provision of counseling, antiretroviral therapy guidelines, clinical and laboratory monitoring and opportunistic infection prophylaxis. ART is supplied either through a central pharmacy which supplies named-patient medication to sites via a courier, or through on-site pharmacies. Clinical data are collected on standardized forms and entered into a central database. Aurum staff provide training, based on the programme guidelines, for clinic staff both prior to programme initiation, and refresher training at intervals afterwards. Individual sites determine where within their health service the programme will be delivered, which pharmacy system is used, and manage clinic staff.

Eligibility for ART is determined using the CD4 count and World Health Organization (WHO) clinical stage, which, at the time of this study, was based on the 1990 definitions [20]: in brief, WHO stage 1 is asymptomatic infection, stage 2 is mild disease such as mucocutaneous manifestations, stage 3 is moderately severe disease but generally not defining AIDS (including pulmonary tuberculosis within the previous year) and stage 4 is severe disease including many AIDS-defining conditions. Individuals are eligible for ART if they have HIV infection and have a CD4 count <250 × 10^6^/l regardless of World Health Organization (WHO) stage of HIV disease; or are in WHO clinical stage 3 with a CD4 count below 350 × 10^6^/l; or are in WHO clinical stage 4, regardless of CD4 count. Prior to ART initiation, ART-eligible individuals have at least two clinic visits and participate in a preparatory three-step counselling process. Standardised drug regimens are used: the first line regimen is zidovudine, lamivudine and efavirenz, and the second line regimen is abacavir, didanosine and lopinavir-ritonavir. Single drug substitutions are made if necessary, for example, stavudine is substituted for zidovudine for patients with anaemia, and nevirapine for efavirenz for women of child-bearing potential. Other changes to the regimens can be made after consultation with the clinical HIV consultants. All ART starts, changes and stops are recorded by the clinician on a form which details the regimen and the reason for the decision.

At every clinic visit, data including self-reported adherence to ART, WHO clinical stage and weight are recorded. Individuals reporting less than perfect adherence are offered additional counseling. In accordance with clinic guidelines, CD4 count and plasma viral load are measured at baseline and at six weeks, six months and every six months thereafter.

A pharmacy register records when ART prescriptions are received and when prescriptions are collected from the pharmacy. If ART is not collected, clinic staff are notified, and the participant is invited back to the clinic for further evaluation and counselling.

### Study design and participants

We analysed virological outcomes on ART using clinic data as an observational cohort. We included HIV-infected adults who were previously ART-naïve, who started ART before 1 July 2004 from nine companies (39 sites) who have at least 10 patients within their HIV care programme. We included follow-up data available up to 31 March 2006: thus all included individuals had the opportunity for at least 12 months of follow-up. The ART start date was defined as the date ART was first prescribed. For individuals who left the programme due to end of employment, death or other reasons, exit dates were determined using treatment stop forms completed by clinic physicians. Individuals who died were not considered as treatment failures, consistent with other reports [21] because most deaths in developing country ART programmes occur soon after ART start [22], before virological failure is likely to have occurred. Individuals who were lost to follow-up during the study were investigated to determine the date of and reason for discontinuation. The main analysis included all individuals with an HIV-1 RNA measurement at 12 months, and individuals who switched to second-line treatment prior to the 12 month visit, who were considered as having virological failure at 12 months. A further analysis also included individuals remaining in the workforce but who had stopped ART prior to the 12 month visit, with these individuals assumed to have virological failure at 12 months. Individuals who had discontinued because they left the workforce were excluded from all risk factor analysis. HIV-1 RNA levels at 6 weeks, 6 and 12 months were defined as the measurement taken between 28–120 days, 121–240 days and 300–450 days following initiation of ART, respectively. If there was more than one measurement in the interval the HIV-1 RNA taken closest to respective visit was used.

### Laboratory protocol

Plasma HIV-1 RNA levels were determined at one central laboratory using the Versant HIV-1 RNA 3.0 assay (bDNA) (Bayer Corporation, Tarrytown, New York, USA.), measurement range 50–500,000 copies/ml. CD4 counts were determined using either FACSCOUNT (BD Biosciences, San Jose, California) or FACSCalibur (BD Biosciences, San Jose, California).

### Data management and statistical analysis

Data were entered into a password protected Access database (Microsoft Office, 2000) and verified. Analysis was carried out using STATA v.9 (Stata Corporation, College Station, Texas, USA).

Plasma HIV-1 RNA concentrations were analysed using the log_10 _scale; values above or below the quantifiable range were assigned values at the respective limits of the assay. Risk factors for suboptimal virological outcome (>= 400 HIV-1 RNA copies/ml) at 12 months were analysed by logistic regression. Risk factors considered were primarily baseline (at start of ART) variables, but we also looked at virological response and self-reported adherence at six weeks, because this is the first point after ART start at which viral load is measured routinely and represents an early point at which interventions to promote adherence could be implemented if required. When analyzing the association with self-reported adherence to ART at the six-week visit, because only about 10% of individuals reported missing any ART, we categorized adherence as perfect (100% reported adherence in last 3 days) and non-perfect (<100%). Factors with a P value of < 0.10 were considered for the multivariable analysis. When considering programme delivery site as a risk factor, sites where greater than 100 individuals had started ART were considered as separate entities (sites A, B and C, each of which serves individuals working for a single company). Sites which were too small to be analyzed separately were grouped; those belonging to one company were considered as site D and all other sites as site E, on the basis that policy differed by company regarding implementation of the ART programme on issues such as pharmacy arrangements and clinic staffing levels. The likelihood ratio test was used to assess associations between risk factors and outcome.

### Ethical considerations

This study was approved by the Research Ethics Committees of Anglogold Health Service, South Africa and the London School of Hygiene & Tropical Medicine, UK.

## Results

**Figure 1:**
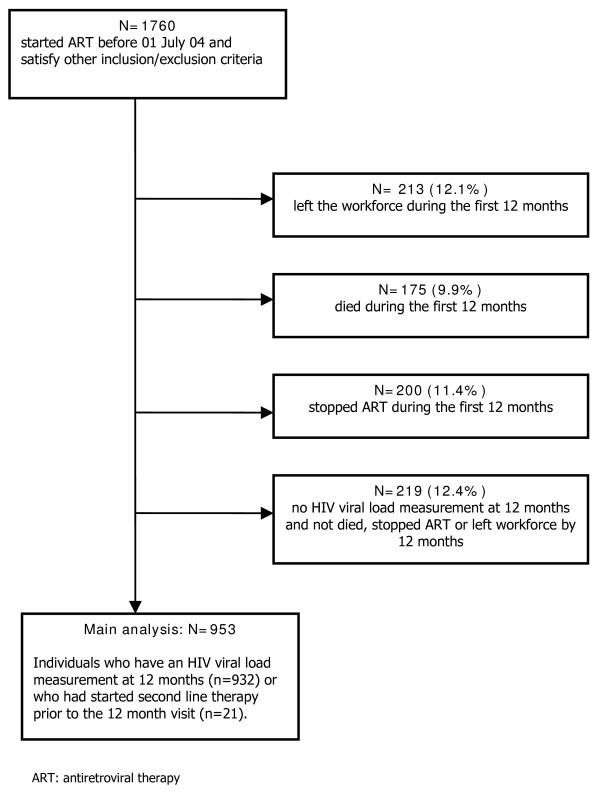
Flow diagram showing cohort construction.

A total of 1760 individuals had started ART before 1st July 2004, satisfied the other inclusion and exclusion criteria and were therefore eligible for the analysis. The median age was 41 years (range 21–66 years) and 97% (1701/1760) were male, reflecting the gender distribution of the workforce. Of the 1760 individuals, 175 (9.9%) and 213 (12.1%) had died or left the workforce before the 12 month follow-up, respectively, and 200 (11.4%) stopped ART before 12 months (Figure 1). Of the remaining 1172 individuals, 81.3% (953/1172) either had a viral load measurement at 12 months or started second line therapy prior to the 12 month visit, and data are subsequently reported for these individuals. Of those in the programme at 12 months, baseline CD4 counts, viral load and 6 week viral loads were similar in the 219 individuals who did not have viral load measured at 12 months compared with the 953 who did have viral load measured at 12 months. The median time from initiation of ART to the 6 week HIV-1 RNA measurement was 7.1 weeks (inter-quartile range [IQR]: 6.3–8.9). For the 6 and 12 month HIV-1 RNA measurements the median times from initiation of ART were 5.9 (IQR: 5.6–6.3) and 12.0 (IQR: 11.4–12.6) months, respectively.

The age and gender distribution of individuals with a viral load result at 12 months was similar to the overall group (median age 41 years, 96% male). At the time of starting ART the median weight was 63 kg (IQR: 57–69 kg), median CD4 count was 156 × 10^6^/l (IQR: 86–230 × 10^6^/l) and 73.0% of individuals were in WHO stage 3 or 4 (Table 1). There were differences in baseline characteristics between sites, notably for WHO stage and CD4 count. At 6 weeks, 6 and 12 months the median (IQR) increases in CD4 count from baseline were 76 (21–142), 92 (28–162) and 107 (33–216) × 10^6^/l, respectively. By 12 months the median gain in weight was 3 kg (IQR: -1 to 7 kg). At 6 weeks 88.6% (669/755) of individuals had a greater than 1 log decrease in viral load, and at 6 weeks, 6 and 12 months 81.2% (651/802), 78.2% (594/760) and 71.5% (681/953) of individuals had virological suppression (defined as viral load < 400 copies/ml), respectively. Of the 656 individuals who had viral load measurements at 6 weeks, 6 and 12 months 63.4% (n = 416) had suppressed viral load levels at all three time points.

**Table 1:** Baseline characteristics of the cohort overall and by ART delivery site

		Overall	By ART delivery site				
		
			Site A	Site B	Site C	Site D	Site E
	
	n	953	165	238	198	145	207
Age (years)	Median	41	38	39	44	39	44
	IQR	35–46	34–41	34–44	39–48	32–46	37–49
Sex	Male (%)	95.9	97.0	97.1	99.0	91.0	94.2
CD4 (× 10^6^/l)	Median	156	177	140	160	131	169
	(IQR)	86–230	106–280	77–205	83–250	88–185	101–222
WHO stage*	1–2 (%)	27.0	16.0	12.8	28.4	38.8	45.7
	3	46.2	36.4	47.7	53.6	45.5	45.7
	4	26.8	47.5	39.6	18.0	15.7	8.7
Viral load (copies/ml)	<= 10,000 (%)	20.1	20.1	20.5	18.1	16.7	24.0
	10,001–100,000	49.7	46.5	50.5	51.3	48.6	50.5
	>100,000	30.3	33.3	29.0	30.6	34.8	25.5
Weight (kg)	Median	63	64	65	61	60	65
	IQR	57–69	59–69	59–70	56–68	52–67	60–73
ART regimen	COM/EFV (%)	94.1	96.4	94.5	97.0	86.9	94.2

ART = antiretroviral therapy; IQR = interquartile range; WHO = World Health Organization; COM = Combivir; EFV = Efavirenz

* clinical staging of HIV disease (see methods for definition).

Table 2 summarises univariable risk factors for a suboptimal virological outcome at 12 months. Older age, higher viral load, lower weight and WHO stage 3 or 4 at baseline, less than a 1 log decrease in viral load at 6 weeks and less than 100% adherence at 6 weeks were all associated with an increased odds of suboptimal virological outcome at 12 months. Large differences in virological response at 12 months were also observed by site of delivery; the percentage with viral load >= 400 copies/ml at 12 months ranged from 18–39% across the five sites.

**Table 2:** Univariable and multivariable associations for suboptimal viral load response (>= 400 copies/ml) at 12 months

		Total	Viral load >= 400 copies/ ml N (%)	Unadjusted Odds ratio	95% confidence interval	P value*	Adjusted Odds ratio	95% confidence interval	P value*
Sex	Male	914	258 (28)	1	-	0.31	1		0.52
	Female	39	14 (36)	1.42	0.73–2.78		1.38	0.53–3.61	

Age – grouped (years)	<35	225	53 (24)	1		0.16	1		0.33
	35–39	199	51 (26)	1.12	0.72–1.74		1.50	0.83–2.71	
	40–44	227	70 (31)	1.45	0.95–2.20	P_t _= 0.01	1.43	0.82–2.52	
	45–49	166	53 (32)	1.52	0.97–2.38		1.89	1.04–3.43	
	>= 50	136	45 (33)	1.60	1.00–2.57		1.50	0.77–2.93	

Site	Site A	165	35 (21)	1	-	<0.001	1		<0.001
	Site B	238	44 (18)	0.84	0.51–1.38		0.71	0.39–1.32	
	Site C	198	78 (39)	2.41	1.59–3.86		2.38	1.32–4.31	
	Site D	145	49 (34)	1.90	1.14–3.15		1.80	0.92–3.50	
	Site E	207	66 (32)	1.74	1.08–2.79		1.70	0.88–3.31	

WHO stage** (n = 55 missing)	1–2	242	52 (21)	1	-	0.02	1		0.007
	3	415	131 (32)	1.69	1.16–2.44		2.08	1.28–3.34	
	4	241	66 (27)	1.38	0.91–2.09		2.03	1.14–3.62	

CD4 count at baseline (× 10^6^/l) (n = 45 Missing)	<= 50	126	37 (29)	1	-	0.5			
	51–100	136	46 (34)	1.23	0.73–2.07				
	101–200	332	95 (29)	0.96	0.61–1.51				
	201–350	273	70 (26)	0.83	0.52–1.33				
	> 350	41	13 (32)	1.12	0.52–2.39				

Viral load at baseline (copies/ml) (n = 61 missing)	<= 10,000	179	36 (20)	1		0.02	1		<0.001
	10,001–	443	134 (30)	1.72	1.13–2.62		3.63	1.88–7.00	
	100,000	270	85 (31)	1.83	1.17–2.85		3.54	1.79–7.00	
	>100,000								

Weight (kg) (n = 77 missing)	<= 50	54	22 (41)	1	-	0.02			
	51–60	285	90 (32)	0.67	0.37–1.22				
	61–70	349	83 (24)	0.45	0.25–0.82	P_t _= 0.007			
	>70	185	46 (25)	0.48	0.25–0.91				

ART regimen	COM/EFV	897	249 (28)	1	-	0.04			
	other	56	23 (41)	1.81	1.04–3.15				

<1 log decrease in viral load at six weeks (n = 198 missing)	No	669	157 (23)	1	-	<0.001	1		<0.001
	Yes	86	40 (47)	2.84	1.79–4.49		4.71	2.56–8.68	

Self-reported adherence at 6 weeks (n = 73 missing)	100%	784	204 (26)	1	-	<0.001	1		<0.001
	<100%	95	43 (45)	2.35	1.52–3.63		3.50	1.92–6.35	

* P-value from likelihood ratio test; P_t _= P-value for linear trend

** clinical staging of HIV disease (see methods for definition).

WHO = World Health Organization; ART = antiretroviral therapy; COM = Combivir; EFV = Efavirenz

The multivariable analysis is reported in Table 2. Virological response and self-reported adherence at six weeks were included in the same model as the data suggests these variables may capture different information (Table 3). Amongst n = 719 patients with both viral load and adherence measured at 6 weeks 18% had differing results; 10% (72/719) reported 100% adherence but had <1 log decrease in viral load, whereas 8% (60/719) reported <100% adherence but had satisfactory virological response. Site of ART delivery, WHO stage, viral load at baseline, unsatisfactory decrease in viral load at six weeks and less than 100% self-reported adherence at six weeks were all independent risk factors for suboptimal virological outcome.

A further analysis was carried out including the 200 individuals who had stopped ART prior to the 12 month visit. Reasons for stopping ART included poor patient adherence or adverse event (63%; n = 126), patient request (11%; n = 23), failure by patient to collect ART prescription (20%; n = 39) and 6% for other reasons. Assuming these additional 200 individuals had suboptimal virological outcome at 12 months 59.1% would have virological suppression at 12 months. Risk factors for poor outcome were similar to the analysis reported in Table 2.

**Table 3:** Cross tabulation of satisfactory virological response at 6 weeks and self-reported adherence at 6 weeks (n = 719)

		Decrease in viral load at six weeks: n (% of overall total)	
	
		≥1 log	<1 log
Self-reported adherence at 6 weeks: n (% of overall total)	100%	578 (80.4)	72* (10.0)
	<100%	60* (8.3)	9 (1.2)

* Discrepant results for virological response at 6 weeks and reported adherence at 6 weeks

## Discussion

The earliest reports from ART programmes in low-income countries were from programmes managed by non-governmental organizations. In their early phases when ART availability was limited, some programmes had stringent entry criteria, requiring good adherence to visits and to preventive therapy before ART was started [7]. These entry criteria are likely to have resulted in a clinic population pre-selected for good adherence. An unintended additional consequence may have been very high mortality among individuals waiting to start treatment [23]. In our programme the criteria for offering ART were medical need, with no prerequisite to demonstrate adherence, making it more similar to routine programmes in industrialized countries. Virological outcomes in those who had a 12 month blood sample taken were similar to those reported from clinic cohorts in the United States [24], but losses from our programme are higher than reported from such cohorts, and this is a limitation of our study.

We can distinguish two main reasons for losses from our programme; individuals who have left the workforce, which may not relate to virological outcome, and those remaining in the workforce who have stopped ART, who may be assumed to have treatment failure. The reasons for default have been briefly reported elsewhere [25] and are currently being investigated in detail. From patient interview the reasons cited for leaving the programme included use of traditional medicines, side effects and not being convinced of the benefits of treatment. A systematic review of patient retention in ART programmes in sub-Saharan Africa found that the percentage of patients retained at 12 months from initiation of ART (where retention was defined as patients known to be alive and receiving ART) varied between 49%–90.3% (median 76%) [26]. Based on this definition patient retention in our study was comparable at 67%.

As ART roll out progresses, retention within programmes will become a key issue determining long-term success. Among patients in our programme who had a viral load measured, 71.5% had suppressed viral load at 12 months following initiation of ART. Using the same analysis approach other South African studies have reported better outcomes [7,9] whilst a small study in Senegal reported 61.9% with viral load < 500 copies/ml at 12 months [10].

We found that high baseline viral load and more advanced WHO stage were risk factors for a suboptimal virological outcome at 12 months. A fall in viral load of less than one log at 6 weeks predicted a suboptimal virological outcome at 12 months, consistent with reports from industrialized countries [15,16,27,28], as did reported less than perfect adherence at six weeks. In our setting there was very little ART use prior to the start of this cohort and hence transmitted resistance among our participants is unlikely; poor virological response is most likely to be due to poor adherence. Suboptimal adherence should be identified as early as possible, so that additional support can be provided, aiming to minimize the risk of ART resistance developing. In resource-limited settings, if it is possible to measure baseline viral load, an early measure of viral load on treatment (for example at four to six weeks) would be arguably more valuable than at the six month visit as suggested, for example, in South African guidelines [29]. Self-reported adherence was also a strong predictor of outcome at 12 months which may be useful if viral load measurements cannot be done.

Our programme provides an unusual opportunity to investigate the influence of health facility level, as well as individual level, factors on ART outcomes. Site was an important determinant of virological outcome which persisted after adjustment for individual factors. Given that all sites used the same clinical guidelines, criteria for starting ART, counseling protocols and drug regimens, it is interesting that site had such a large effect on the probability of achieving virological suppression. With relatively few large sites to compare we cannot formally investigate at the health facility level which may influence virological outcomes. However our experience suggests some factors which may be important include long waiting times for clients (both in clinics and in pharmacy); using staff who do not have specialized training in HIV care; failure to contact individuals who do not attend clinic and lack of communication between pharmacy and clinic staff if ART is not collected. We plan to explore these issues in further work, including comparison of costs at different clinic sites.

## Conclusion

In conclusion, in this cohort of adults starting ART in South Africa, the strongest independent predictors of virological outcome at 12 months were the decrease in viral load and self-reported adherence at six weeks. In settings with limited access to viral load measurements, the virological response would be more usefully determined at this point rather than later, so that appropriate interventions can be implemented. Self-reported adherence at six weeks was also an independent predictor of outcome. Health system factors have an important influence on virological outcomes; this needs further investigation to guide implementation of effective ART programmes.

## Competing interests

The authors declare that they have no competing interests.

## Authors' contributions

KLF, SC, ALS, GJC, ADG designed the study. SC, ALS, LFP, DJM, RW collected the data. KLF, ALS, ADG carried out data analysis and interpretation. SC and GJC carried out data interpretation. KLF, SC, ALS, ADG drafted the manuscript. LFP, DJM, RW, GJC revised the manuscript. All authors have read and approved the final version of the manuscript.

## Pre-publication history

The pre-publication history for this paper can be accessed here:


